# Potential Barriers to Healthcare in Malawi for Under-five Children with Cough and Fever: A National Household Survey

**Published:** 2014-03

**Authors:** Marte Ustrup, Bagrey Ngwira, Lauren J. Stockman, Michael Deming, Peter Nyasulu, Cameron Bowie, Kelias Msyamboza, Dan W. Meyrowitsch, Nigel A. Cunliffe, Joseph Bresee, Thea K. Fischer

**Affiliations:** ^1^Department of Public Health, Faculty of Health Sciences, University of Copenhagen, Copenhagen, Denmark; ^2^Department of Community Health, College of Medicine, University of Malawi, Blantyre, Malawi; ^3^Centers for Disease Control and Prevention, Atlanta, GA, USA; ^4^Division of Epidemiology and Biostatistics, School of Public Health, Faculty of Health Sciences, University of the Witwatersrand, Johannesburg, South Africa; ^5^Department of Clinical Infection, Microbiology and Immunology, Institute of Infection and Global Health, University of Liverpool, Liverpool, United Kingdom

**Keywords:** Healthcare surveys, Health expenditure, Health services accessibility, Malaria, Pneumonia, Malawi

## Abstract

Failure to access healthcare is an important contributor to child mortality in many developing countries. In a national household survey in Malawi, we explored demographic and socioeconomic barriers to healthcare for childhood illnesses and assessed the direct and indirect costs of seeking care. Using a cluster-sample design, we selected 2,697 households and interviewed 1,669 caretakers. The main reason for households not being surveyed was the absence of a primary caretaker in the household. Among 2,077 children aged less than five years, 504 episodes of cough and fever during the previous two weeks were reported. A trained healthcare provider was visited for 48.0% of illness episodes. A multivariate regression model showed that children from the poorest households (p=0.02) and children aged >12 months (p=0.02) were less likely to seek care when ill compared to those living in wealthier households and children of higher age-group respectively. Families from rural households spent more time travelling compared to urban households (68.9 vs 14.1 minutes; p<0.001). In addition, visiting a trained healthcare provider was associated with longer travel time (p<0.001) and higher direct costs (p<0.001) compared to visiting an untrained provider. Thus, several barriers to accessing healthcare in Malawi for childhood illnesses exist. Continued efforts to reduce these barriers are needed to narrow the gap in the health and healthcare equity in Malawi.

## INTRODUCTION

Worldwide, an estimated 8.8 million children aged below five years die annually. Acute respiratory infection is the primary cause of these deaths, accounting for 18% of under-five mortality, followed by diarrhoea and malaria (1). The vast majority of child deaths occur in developing countries, and health inequities are not only widespread across countries but also within countries. Children from the poorest households are more likely than children from wealthier households to be exposed to health risks, to be malnourished, to experience reduced access to preventive and curative healthcare services and, consequently, to die in childhood (1-5).

Adequate access to and utilization of healthcare services are crucial to improve child health in developing countries (2,3). However, the rate of obtaining care from a trained healthcare provider remains low in many developing countries; instead, children are often treated at home, by an untrained care provider, or not treated at all (2-4). Studies have demonstrated that multiple barriers to healthcare exist. Geographic accessibility of facilities is a key determinant of utilization, and factors, such as rural residency, long distance, and high travel costs, have been shown to reduce accessibility (6-8). Economic affordability is another major determinant, and there is ample evidence that low household income and high care-seeking costs are barriers to healthcare (6-10). In addition, a wide range of demographic factors have been identified to affect utilization of services, including age, sex, educational level, ethnicity and religion, socioeconomic status, and family-size and composition (6-8,10). Finally, cultural attitudes and beliefs of the population influence their healthcare utilization patterns (6-8). There is a need for analyzing the interrelation between the different restrictive factors in more detail (6).

The healthcare delivery system in Malawi is three-tiered, consisting of primary, secondary, and tertiary-care levels. Sixty-eight percent of health services are provided by the public sector and 32% by the private sector, including the not-for-profit Christian Health Association of Malawi (CHAM). In addition, traditional healers are widely used (11,12). To ensure effective services and equitable access, the Government of Malawi implemented an Essential Health Package (EHP) in 2002 and launched a sector-wide approach in 2004 as the vehicle to deliver EHP services which are provided free of charge (13). However, the healthcare system has been constrained in the provision of services in recent years due to a number of factors (11). The financial resources for health service delivery have been inadequate and unpredictable. Consequently, Malawi's EHP services have been underfunded since its implementation (11,14). Furthermore, there has been an increasing shortage of medical staff since 1990 as a result of poor working conditions, low wages, deaths caused by the HIV/AIDS epidemic, and migration of skilled personnel to developed countries (11,15). Quality of care has been further compromised by a periodic stock-out of essential drugs and medical supplies (11). Despite these challenges, Malawi has achieved significant reductions in the infant and under-five mortality rates, with an annual average decline of 4.3% and 4.7% respectively during the period 1990-2011 (16). Key factors responsible for this progress include consistent investment in child survival interventions and strong coordination between the Government of Malawi and the development partners (17). However, while national averages have improved, the poorest children have benefited the least from this progress (18). The annual average reduction rates in infant and under-five mortality for the poorest 20% of children have been respectively 2.2% and 2.7% during the same period (18). Thus, pro-poor targeting has not yet resulted in equal progress in mortality reduction across socioeconomic strata. Consequently, inequities in health and access to healthcare disfavouring the poor have persisted and widened (5,17,18). This situation may potentially affect Malawi's progress towards the United Nations’ Millennium Development Goal to reduce child mortality (17).

To take appropriate measures towards improved and equitable access to healthcare services, the determinants of limited access need to be identified in the specific context of Malawi (2,3,18,19). The overall aim of the present study was to assess potential barriers to healthcare in Malawi for children aged below five years, with cough and fever. We studied the interrelated effects of demographic and socioeconomic predictors of obtaining care from trained providers. Furthermore, we estimated the direct and indirect costs of seeking care, thereby providing detailed cost data which are sparse in the literature (6,9).

## MATERIALS AND METHODS

### Study site and population

The Republic of Malawi is a small sub-Saharan African country bordering Zambia and Tanzania to the north and partly embedded into Mozambique to the south. The total population in 2011 was 15.4 million (20). Malawi is among the poorest and the least developed countries in the world, ranking 171 out of 187 countries in the Human Development Index (20). Eighty percent of the population lives in rural areas, and approximately 74% lives below the poverty line and 40% in severe poverty (20). The life expectancy at birth is 54.2 years, and the infant and under-five mortality rates are 53 and 110 per 1,000 livebirths respectively (20).

The transmission of malaria is perennial in Malawi, with a peak in the rainy season from November to April (21). The transmission of acute respiratory infections peaks in the beginning of the cold dry season in April to June (22).

### Study design and data collection

We conducted a national household survey from the end of February to mid-April 2005, using a cluster-sample design with compact segments (23,24). We aimed for a nationally-representative sample of 600 children, equaling 3,000 households. Sample-size calculation took into account the expected proportion of interest (i.e. the proportion of children with cough and fever seeking care from a trained healthcare provider), the level of precision desired, and the expected design effect (23). Malawi's 1998 Population and Housing Census provided a complete list of enumeration areas (EAs) (25). The projected number of households in each EA was calculated based on census data, then divided by 100 and rounded to the nearest integer, which was considered to be the size of the EA in segments. This predetermined segment-size was based on the prediction that the survey team could complete interviews in 100 households per day. Subsequently, 30 EAs were chosen by systematic sampling with probability of selection proportional to the size of each EA in segments (23). When the survey team arrived at an EA, it was divided into the predetermined number of segments, using sketch maps, such that each segment in the EA had approximately the same number of households. Among these segments, one segment was then selected at random (23). All households in the segment were visited by interviewers who interviewed all primary caretakers of children aged less than five years; if found absent, households were revisited later the same day. Data were entered into Personal Digital Assistants (PDAs), using Visual Basic^®^ software (version 6) (Microsoft Corporation, Redmond, WA, USA) and analyzed with the SAS-callable version of SUDAAN^®^ software (version 9.01) (Research Triangle Institute, Research Triangle Park, NC, USA) to take account of the cluster-sampling design. Data collection was performed by one survey team of 6 interviewers and 2 supervisors.

**Figure 1.**
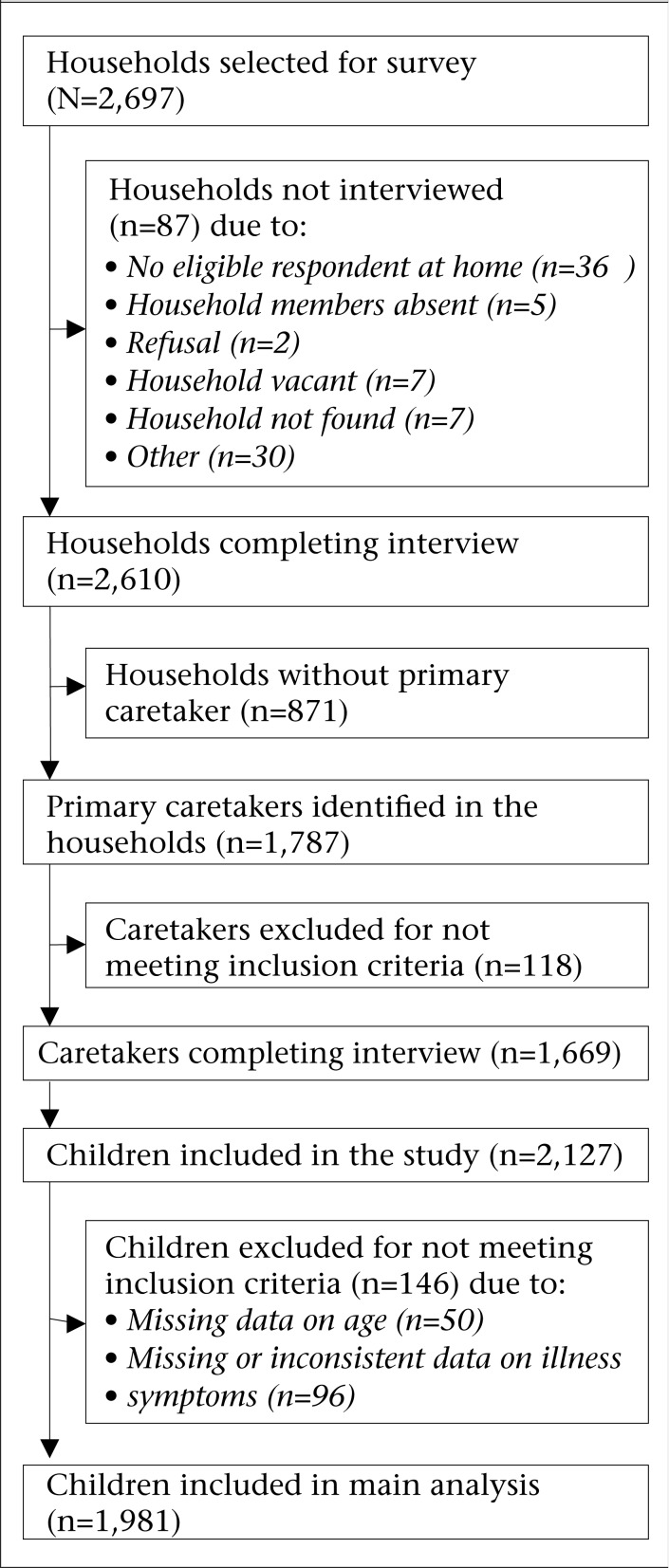
Flowchart of selecting participants

In total, 2,697 households were selected for the survey and visited by survey teams. Figure 1 shows a flowchart of inclusion and exclusion of study participants. Interviews were completed in 2,610 (96.8%) of the households; reasons for non-completion are listed in Figure 1. Among the surveyed households, 871 households had no primary caretaker. Thus, a total of 1,787 primary caretakers were identified; 1,669 (93.4%) of them were eligible for the survey, i.e. had at least one child below five years of age and were interviewed.

The first part of the questionnaire recorded the demographic and socioeconomic characteristics of the household, including access to safe water, and was completed by all caretakers identified for the survey (26). The second part focused on illness symptoms and healthcare utilization among children aged below five years. This part was only completed if the child had cough and fever during the two weeks preceding the survey, which was determined by a screening question in the beginning of Part II of the questionnaire. A third part of the questionnaire was completed if the child had diarrhoea during the two weeks preceding the survey

### Variables

**Figure 2.**
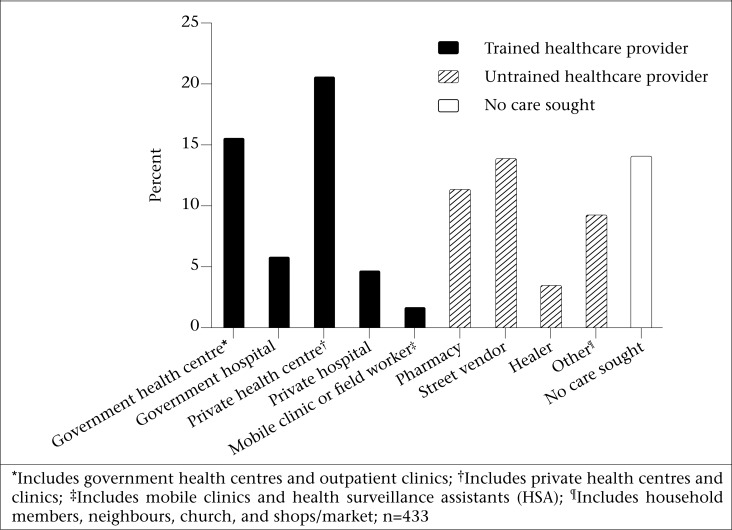
Distribution of healthcare providers visited among children below five years of age, with cough and fever, Malawi 2005

*Healthcare utilization (dependent variable):* We identified 12 different types of healthcare providers in Malawi and defined healthcare utilization as primary visits to any of those. Providers were categorized as being trained or untrained based on whether or not they held a formal medical degree or medical qualifications. Trained providers were further categorized as working in private or government healthcare facilities (Figure 2).

*Age of children:* The following 5 age categories were used: <12, 12-23, 24-35, 36-47, and 48-59 completed months. In the analyses, age categories were dichotomized into <12 months and 12-59 months of age due to statistical uncertainty around the small number of subjects in 5 categories.

*Education of mother:* Mothers were asked if they had ever attended school, with ‘yes/no’ answers.

*Wealth index:* We defined socioeconomic status in terms of assets. Data on household assets were combined into a wealth index by principal components analysis. Households were assigned a standardized score for each asset, such as drinking-water source, type of toilet facility, electricity, and vehicles, according to a Malawi-specific index (27). The scores were summed for each household. Households were ranked and divided into terciles. The distribution of the summed up asset scores was right-skewed, i.e. the bulk of households had asset scores below the mean value. Consequently, if divided into quintiles, the lowest wealth quintiles would only slightly differ from one another in terms of asset scores, and a significant number of households with identical asset scores would group into different wealth quintiles due to the heaping of scores. Therefore, we opted to divide the sample into terciles. In the analyses, wealth quintiles were dichotomized into the poorest (the two lower terciles) and the wealthiest (the upper tercile) due to statistical uncertainty around the small number of subjects in three categories.

*Other demographic variables:* Child's sex (male/female) and place of residence (urban/rural) were also included.

*Severity of illness:* In our screening question, we identified children who had cough and fever during the two weeks preceding the survey. Additional symptoms, reported by caretakers, were collected only for children who had both of these symptoms. Malawi's Ministry of Health has adopted the guidelines of the World Health Organization's Integrated Management of Childhood Illness (IMCI) for the case management of childhood illnesses at the first-line health facilities (28). We applied the IMCI classifications, with modifications, to categorize the symptoms of illness. We defined two illness categories each comprising two subcategories: moderately-severe illness comprising “malaria without pneumonia” and “malaria with pneumonia”; and severe illness comprising “severe pneumonia or very severe febrile disease”, and “possible serious bacterial infection.” Children aged 2 months up to 5 years with cough and fever without any danger signs were classified as having “malaria without pneumonia”, and children with cough and fever accompanied with fast breathing but without any danger signs were classified as having “malaria with pneumonia.” Children aged 2 months up to 5 years, with cough and fever and any of the following danger signs: being unable to eat or drink, convulsions, lethargy, unconsciousness, and/or stiff neck, were classified as having “severe pneumonia or very severe febrile disease.” Children aged 1 week up to 2 months, with cough and fever, were classified as having “possible serious bacterial infection.”

*Direct and indirect costs:* Costs associated with seeking care were measured in both monetary terms, i.e. direct costs, and in terms of time, i.e. indirect costs. The direct costs included travel costs, user fees for consultation and/or hospitalization, and the costs of drugs. Direct costs were measured as the sum of out-of-pocket payments in local currency—the Malawi Kwacha (MWK). The official exchange rate in 2012 was 1 US$=269.5 MWK (29). Indirect costs were measured as time spent travelling and waiting when seeking care. The opportunity cost, i.e. the costs of activities forgone due to time spent on seeking care, was assumed to be lost income. Hence, time spent on seeking care was transformed into lost income per minute as its monetary equivalent, using income data from the Malawi Integrated Household Survey (30). Waiting time was assumed to be 75 minutes for all caretakers, in accordance with other studies (31).

### Ethical approval

The study received ethical approval from the Ethics Review Committees of the College of Medicine, Malawi and the Centers for Disease Control and Prevention, Atlanta, GA, USA, prior to the enrollment of participants. Informed consent was obtained by the research assistants for each participant prior to his/her enrollment in the study. Informed consent for this study conformed to the 45 Code of Federal Regulations, Part 46.101c and 46.102d requirements.

### Statistical analyses

We used univariate analyses to assess the effect of the six predictive variables (child's age, sex, education of mother, wealth index, place of residence, and illness severity) on seeking care from a trained healthcare provider compared to seeking care from an untrained provider or not seeking care at all. Only primary healthcare visits were included and, thus, successive visits were excluded from analysis. Odds ratios (ORs) and 95% confidence intervals (95% CI) were calculated for each variable, and the p value of a Wald F-test was evaluated to determine whether associations were significant (p<0.05).

All six variables and all two-factor interaction terms were entered into a multiple logistic regression model. This model was reduced to the final model by means of a hierarchical backward elimination procedure. Significance was determined by evaluating the p value of Wald F-test (p<0.05). If an independent variable was not significant but, when removed, it changed the OR of a significant variable by ≥10%, and it was retained in the model to adjust for confounding effect; *t*-tests were used for comparing means in travel time and direct costs incurred when seeking care with regard to wealth index, place of residence, and type of healthcare provider visited. Furthermore, we calculated the total cost each caretaker incurred when seeking care and investigated total cost relative to income as described elsewhere (31).

## RESULTS

Caretakers provided information on 2,127 children (Table 1). The sample of children obtained was representative of the child population in Malawi, according to the 2004 Malawi Demographic and Health Survey, with regard to sex and place of residence (data not shown) (32). A total of 50 children were excluded from further analysis due to missing data on age (Figure 1). Among the remaining 2,077 children, 504 (24.3%) experienced one episode of cough and fever in the two weeks preceding the survey, and 28 (1.3%) experienced two episodes, resulting in a total of 532 illness episodes. Illness could not be classified in 96 children due to incomplete or inconsistent questionnaire, and these children were excluded from subsequent analyses. In total, 1,981 (93.1%) children were included in the main analyses and, among these children, 433 episodes of cough and fever (in 408 children) had occurred in the two weeks preceding the survey. Of these episodes, 58.0% were classified as moderately-severe illness (47.8% with “malaria without pneumonia” and 10.2% with “malaria with pneumonia”), and 42.0% were classified as having severe illness (39.7% with “severe pneumonia or very severe febrile disease” and 2.3% with “possible serious bacterial infection”).

**Table 1.** Baseline characteristics of surveyed under-five children, Malawi 2005

Variable	Household survey (N=2,127)		
	No. (%)	95% CI
Child's age (completed months)		
<12	440 (21.2)	19.2-23.4
12-23	429 (20.7)	18.9-22.5
24-35	420 (20.2)	18.3-22.3
36-47	316 (15.2)	14.0-16.6
48-59	472 (22.7)	20.8-24.8
Child's sex		
Male	1,043 (50.1)	48.0-52.1
Female	1,040 (49.9)	47.9-52.0
Education of mother		
No	654 (43.9)	36.8-51.3
Yes	835 (56.1)	48.7-63.2
Wealth index		
Poorest	1,340 (68.9)	58.3-77.7
Wealthiest	606 (31.1)	22.3-41.7
Place of residence		
Urban	200 (9.4)	2.6-28.5
Rural	1,927 (90.6)	71.5-97.4

CI=Confidence interval

### Healthcare utilization patterns

A trained healthcare provider was visited for 48.0% (n=208; 95% CI 41.0-55.1) of the illness episodes that had occurred among the surveyed children in the two weeks preceding the survey. An untrained provider was visited for 37.9% (n=164; 95% CI 31.2-45.1) of illness episodes. The remaining 14.1% (n=61; 95% CI 10.7-18.4) of illness episodes did not result in a healthcare visit. The distribution of healthcare providers visited is illustrated in Figure 2. The most common providers worked in private health centres (n=60; 13.9%; 95% CI 10.0-18.8), were street vendors (n=60; 13.9%; 95% CI 10.0-18.8), or worked in pharmacies (n=49; 11.3%; 95% CI 8.3-15.2).

In univariate analyses, child's age and wealth index of the household were associated with seeking care from a trained healthcare provider compared to seeking care from an untrained provider or not seeking care at all (Table 2). Children aged 12-59 months (OR 0.52; 95% CI 0.30-0.90) and children living in the poorest households (OR 0.51; 95% CI 0.30-0.87) were less likely to be taken to a trained healthcare provider when ill compared to older children and those living in wealthier households respectively.  Children with moderately-severe illness (OR 0.72; 95% CI 0.47-1.12) were less likely than children with severe illness to visit a trained healthcare provider; however, this association was not statistically significant.

In the multiple logistic regression model, child's age and wealth index continued being associated with seeking care from a trained healthcare provider (Table 2). The effect of potential confounding variables was assessed, and no confounders were found. Similarly, no significant interactions were observed.

### Direct and indirect costs

Results of *t*-tests demonstrated that time spent travelling when seeking care was significantly associated with place of residence and type of healthcare provider visited (Table 3). Children living in rural areas spent more time travelling than those in urban areas (mean: 68.9 minutes vs 14.1 minutes) when visiting any type of healthcare provider. In addition, children who visited a trained healthcare provider spent more time travelling than those visiting an untrained provider (mean: 76.8 minutes vs 26.2 minutes).

Furthermore, living in the wealthiest households, living in an urban area, and obtaining care from a trained healthcare provider and/or from private healthcare facilities were significantly associated with higher direct costs (Table 3). However, when estimating total cost relative to income, caretakers living in rural areas spent a higher proportion (3.2%) of their monthly income when seeking care than caretakers living in urban areas (3.0%). This result was not significant (Table 4).

To determine the representativeness of children for whom cost data were available, we compared those with children for whom data were missing by using chi-square tests. Our results indicated that a higher proportion of children for whom data on travel time were available was living in the wealthiest households (52.5%; p=0.04) and was taken to a trained healthcare provider (65.4%; p<0.001) compared to children who had missing data on travel time (47.5% and 34.6% respectively). Furthermore, a higher proportion of children who had data on direct costs had an educated mother (56.6%; p<0.01) whereas a lower proportion was taken to a trained healthcare provider (48.1%; p<0.001) compared to children who had missing data on direct costs (43.4% and 51.9% respectively).

**Table 2.** Univariate and multiple logistic regression analyses for seeking care from a trained healthcare provider among under-five children with cough and fever, Malawi 2005

Variable	Total (n=433)*	Total (n=433)†	Univariate analyses		Multiple logistic regression	
			Crude OR (95% CI)	p value	Adjusted OR (95% CI)	p value
Child's age (months)						
<12 (ref)	85	52 (61.1)	1.00		1.00	
12-59	348	156 (44.8)	0.52 (0.30-0.90)	0.02	0.52 (0.30-0.90)	0.02
Child's sex						
Male (ref)	197	97 (49.2)	1.00			
Female	234	111 (47.4)	0.93 (0.55-1.57)	0.78		NS
Education of mother						
No	194	92 (47.4)	0.99 (0.58-1.69)			
Yes (ref)	235	112 (47.7)	1.00	0.97		NS
Wealth index						
Poorest	255	112 (43.9)	0.53 (0.32-0.88)		0.51 (0.30-0.87)	
Wealthiest (ref)	141	84 (59.6)	1.00	0.02	1.00	0.02
Place of residence						
Urban (ref)	41	23 (56.1)	1.00			
Urban (ref)	41	23 (56.1)	1.00			
Rural	392	185 (47.2)	0.70 (0.27-1.78)	0.44		NS
Severity of illness
Moderate	251	112 (44.6)	0.72 (0.47-1.12)	0.14		NS
Severe	182	96 (52.7)	1.00			

*All illness episodes in the two weeks preceding the survey;

†Illness episodes for which care was sought from a trained healthcare provider;

CI=Confidence interval;

NS=Not significant;

OR=Odds ratio;

Ref=Reference group

**Table 3.** Results of *t*-test of travel time and direct costs associated with seeking care for under-five children with cough and fever, Malawi 2005

Variable	Travel time (min) (n=187)*			Direct costs (MWK)† (n=214)‡		
No.	Mean (range)	p value	No.	Mean (range)	p value
Wealth index						
Poorest	107	72.1 (1.0-660.0)	0.08	137	41.6 (0.0-640.0)	0.01
Wealthiest	74	47.9 (1.0-360.0)		70	117.5 (0.0-900.0)	
Place of residence						
Urban	20	14.1 (1.0-60.0)	<0.001	22	177.6 (0.0-600.0)	0.02
Rural	167	68.9 (1.0-660.0)		192	54.2 (0.0-640.0)	
Type of provider						
Untrained	51	26.2 (1.0-120.0)	<0.001	114	34.1 (0.0-500.0)	<0.001
Trained	136	76.8 (1.0-660.0)		100	104.4 (0.0-900.0)	
Type of facility						
Government	63	59.4 (1.0-360.0)	0.09	48	19.7 (0.0-240.0)	<0.001
Private	73	91.8 (1.0-660.0)		52	182.5 (0.0-900.0)	

*Illness episodes for which information on travel time were available;

†Direct costs include travel costs, user fees, and costs of drugs measured in local currency—the Malawi Kwacha;

‡Illness episodes for which information on direct costs were available;

Min=Minutes; MWK=Malawi Kwacha

**Table 4.** Total costs of seeking care relative to monthly household income for under-five children with cough and fever, Malawi 2005

Variable	Number	Monthly household income(MWK)*	Direct costs (MWK)†	Travel time (min)	Waiting time (min)‡	Total time spent (min)¶	Total time costs (MWK)§	Total costs (MWK)**	% of monthly income
Urban	11	10,784	213.2	19.0	75.0	94.0	105.2	318.4	3.0
Rural	115	3,353	57.8 p<0.05	70.5 p=0.1	75.0	145.5 p=0.1	50.9 p=0.08	108.7 p<0.05	3.2 p=0.5

*Data from the Malawi Integrated Household Survey (30) measured in the local currency—the Malawi Kwacha;

†Direct costs include travel costs, user fees, and costs of drugs measured in the local currency—the Malawi Kwacha;

‡Waiting time was assumed to be 75 minutes for all caretakers in accordance to other studies (31);

¶ Sum of travel time and waiting time;

§ Average cost per minute (calculated as lost income per minute: monthly household income divided by four 40-hour work weeks) multiplied by total time spent;

**Sum of direct costs and total time costs.

MWK=Malawi Kwacha;

Min=Minutes

## DISCUSSION

Our study identifies several potential barriers to obtaining healthcare from a trained healthcare provider in Malawi and thereby refines our understanding of the gravity of these barriers and how they interrelate. This knowledge is important for policy-makers and programme managers in their planning of equitable delivery of healthcare service and child survival interventions in Malawi.

Overall, we found that nearly half of the children with cough and fever were taken to a trained healthcare provider. This rate is considerably higher than rates reported previously in Malawi and Tanzania (32,33). However, this comparison is not straightforward because of different case definitions. In addition, we demonstrated significant inequities in healthcare utilization. We found that infants were two times more likely than older children to be taken to a trained healthcare provider, suggesting that caretakers in Malawi may be particularly aware of the vulnerability of infants with cough and fever. These findings are similar to those reported in other studies (18,32,33). In addition, our results confirmed what has been noted previously in developing countries that children living in the poorest households are less likely to obtain care from a trained healthcare provider when ill (6,9). Caretakers from the poorest households may not be well-informed about danger signs of illness and the availability of effective treatment. They may also be hindered by long distances to health facilities and by financial barriers (2,3,18,33). Consequently, inequities in healthcare are still evident in Malawi. In contrast to other studies, we found no association between maternal education and healthcare utilization, possibly because of dichotomous definition of education in our study (3,6).

A substantial proportion of children was taken to an untrained healthcare provider, particularly to street vendors and retail pharmacy workers. These sources of treatment are convenient in terms of proximity and are relatively low-cost (19,34). However, studies have raised doubt about the quality of drug dispensing, such as the provision of ineffective drugs and stock-outs of drugs (19,35-38).

Our findings further showed that caretakers from rural households spend more time travelling than those in urban areas when seeking care. This is supported by similar studies conducted in Malawi and Ghana (6,39). Rural areas are often underserved by health facilities and a lack of transport and poor road conditions make it even more difficult to travel long distances (6,39). Travel time could be very costly in terms of opportunity costs, primarily lost income (31,39). Long and difficult travel may not only cause delays in healthcare visits and thereby aggravate the illness, it might also hinder children from receiving care (6,31,39).

As expected, the direct costs of seeking care increased in absolute terms for the wealthiest and urban households. When measuring total cost relative to income, caretakers living in rural areas compared to urban areas spent a higher percentage of their monthly income when seeking care. However, this result was not significant. Studies in Zambia and Burkina Faso have reported even more regressive healthcare expenditure than those in our study (9,31). Our estimations of time costs were based on an average income per year. It has previously been reported that healthcare visits in a farmer's high season are even more costly in terms of lost income (31). Healthcare costs often put a major burden on households and, to cope with payments, households adopt strategies, such as the sale of assets and borrowing money. These coping strategies can be catastrophic and may force households into debt or poverty or push them into deeper poverty (40,41). Furthermore, the direct costs were considerably higher when care was sought from a private compared to a government facility. Thus, despite the significant role of the private sector in service provision in Malawi, this sector puts a major economic burden on households seeking care from private facilities.

### Implications

Socioeconomic inequities in healthcare are still evident in Malawi and, therefore, addressing barriers to healthcare is a key priority for improving child health and achieving the Millennium Development Goal to reduce child mortality (18). One of the first responses should be improved access of the poorest households to healthcare services. This could be accomplished by reducing logistical and financial barriers. Geographic availability of healthcare facilities could be improved through targeted interventions, increased availability of health facilities in rural areas, and improved infrastructure (2,6,18,31). This has been recognized by the Government of Malawi, which increased the number of rural health centres from 219 to 258 between 2003 and 2010 (11). Furthermore, basic training of untrained healthcare providers might ensure higher standards of care and referral from these sources. In addition, financial access to care from the private health sector could be improved by reducing costs. This could be achieved by implementation of a fair user-fee system where the poorest people are guaranteed access and treatment through a waiver system, health insurance programmes, or payment by installments (36,43). Since 2005, the Government of Malawi has sought to reduce financial barriers to healthcare by introducing service-level agreements with the CHAM's facilities to allow free access to maternal and child health services. By 2010, seventy-two of 150 private facilities had such free access (11). Other priorities could be community-based interventions to improve healthcare-seeking patterns. Health communication efforts could reinforce caretakers’ ability to perceive the severity of illness and initiate the appropriate response (2,19,38). Finally, it should be emphasized that multisectoral strategies to reducing poverty and socioeconomic inequities are crucial to the success of specific interventions as recognized by the Government of Malawi (2,36).

Future research on healthcare performance by different care providers in Malawi would improve our evaluation of the likely benefits or hazards of observed utilization patterns. Continued research on the trends in healthcare utilization will help assess the success of the initiatives made by the Government of Malawi and the development partners.

### Limitations

Our study had a number of limitations. The cross-sectional study design precluded the identification of a causal relationship between predictor and outcome variables. In addition, relying on retrospective self-reports increased the susceptibility to recall bias and social desirability bias. A two-week recall period was used in minimizing the problem of recall. Our illness classifications were presumptive and, as shown elsewhere, some symptoms are not recognized by caretakers, which may have led to misclassification (33,42). It should also be noted that we only studied a subset of pneumonia and malaria episodes according to the IMCI guidelines, namely those with cough and fever. Another limitation was that the study was conducted during the rainy season in Malawi and, thus, the prevailing illness and healthcare utilization patterns could be different from the dry season. Finally, missing of data in the cost analyses was a limitation of our study, and children for whom cost data were available were not representative of all children. Therefore, our cost results should be interpreted with caution.

### Conclusions

We demonstrated that, in a poor African country, like Malawi, significant inequities exist in obtaining healthcare from a trained provider for childhood illnesses and in the costs associated with seeking care. Continued efforts to reduce these barriers are needed to narrow the gap in health and healthcare equity in Malawi.

## ACKNOWLEDGMENTS

We are grateful to the field team members who interviewed the participants. We thank Roger I. Glass, Centers for Disease Control and Prevention, for his contribution in the planning of our study.
